# Location Prediction for Tweets

**DOI:** 10.3389/fdata.2019.00005

**Published:** 2019-05-24

**Authors:** Chieh-Yang Huang, Hanghang Tong, Jingrui He, Ross Maciejewski

**Affiliations:** CIDSE, Arizona State University, Tempe, AZ, United States

**Keywords:** data mining, location prediction, multi-head self-attention mechanism, joint training, deep learning, tweets

## Abstract

Geographic information provides an important insight into many data mining and social media systems. However, users are reluctant to provide such information due to various concerns, such as inconvenience, privacy, etc. In this paper, we aim to develop a deep learning based solution to predict geographic information for tweets. The current approaches bear two major limitations, including (a) hard to model the long term information and (b) hard to explain to the end users what the model learns. To address these issues, our proposed model embraces three key ideas. First, we introduce a multi-head self-attention model for text representation. Second, to further improve the result on informal language, we treat subword as a feature in our model. Lastly, the model is trained jointly with the city and country to incorporate the information coming from different labels. The experiment performed on W-NUT 2016 Geo-tagging shared task shows our proposed model is competitive with the state-of-the-art systems when using accuracy measurement, and in the meanwhile, leading to a better distance measure over the existing approaches.

## 1. Introduction

Nowadays, many technology systems (e.g., a social media platform) emit a variety of digital information, such as texts, times, logs, and so on. Geographic information has been receiving much attention lately. In fact, there are a large amount of applications benefiting from geographic information, ranging from marketing recommendation systems (Bao et al., 2012; Savage et al., 2012; Yin et al., 2013; Cheng and Shen, 2014) to event detection systems (Sakaki et al., 2010, 2013; Watanabe et al., 2011; Li et al., 2012a). Although technology that allows the user to share his/her geographic information has matured, many users are reluctant to do so due to various concerns such as inconvenience, privacy and so on. As Sloan et al. (2013) illustrated, only <1% of tweets have a geographic tag attached which in turn limits the growth of related applications. Therefore, researchers have tried to automatically identify the location of the user or post on social media sites. In this paper, we target our location prediction problem on Twitter, which is one of the largest social media sites.

Our proposed model takes three concepts into account, multi-head attention mechanism, subword feature, and joint training technique. The first two parts are proposed for better modeling the text representation and the joint training part is related to the whole architecture of our model. In this work, we mainly focus on using only text information instead of other metadata provided by Twitter since our goal is to develop a generic social media location prediction method, which could be further applied to other platforms (e.g., online news where the user information is usually not available). As a result, we introduce different methods to enhance the text representation part as well as the whole model architecture.

Text representation, however, is one of the most important tasks for Natural Language Processing (NLP) applications. In recent studies with the help of deep learning techniques, many NLP tasks, including text classification problem, question answering problem, sentiment analysis, translation, start from designing a good module for capturing useful and meaningful text information. One of the well-known approach is the Recurrent Neural Network (RNN) based models such as vanilla Recurrent Neural Network (vanilla RNN), Long Short-term Memory Neural Network (LSTM), and Gated Recurrent Unit Network (GRU). Some existing works have shown the RNN-based models' power of handling the language modeling tasks (Bengio et al., 2003; Mikolov et al., 2010; Sundermeyer et al., 2012). In RNN, the model iterates all of the text step-by-step and at the same time propagates the information to the next step to form the sentence representation. RNN has achieved a big success in many applications. However, the RNN-based model usually suffers from the extremely long training time because every word depends on all the previous words which makes it hard to parallelize and accelerate. Another branch of studies focuses on the Convolutional Neural Network (CNN) based models (Kim, 2014). Though CNN was originally proposed to solve problems on images, Kim (2014) successfully introduced it into the NLP field. The idea of CNN is to capture some specific n-gram patterns of the text by using lots of filters with various lengths. However, due to the length limitation of the kernel filters, CNN works better in modeling the local information. Therefore, we instead adopt the multi-head self-attention model (Vaswani et al., 2017) to model the text information. Multi-head self-attention model (Vaswani et al., 2017) utilizes only attention mechanism, yet it enjoys the advantages of both RNN and CNN. That is to say, we can perform parallel computing for all text at the same time, and in the meanwhile, long term information is also encoded.

The subword feature was shown to be very useful for tasks built on social media since people tend to use lots of informal language on social media (Zhang et al., 2015; Vylomova et al., 2016). One simple but common example is the use of “Good” with a various number of “o” which produces words like “Goooooood”. Another example is the user-created word such as “Linsanity” which is the combination of “Jeremy Lin” (an NBA player) and “insanity.” Therefore, if we start from subword feature such as character, we could potentially infer the subtle meaning of these words. Many applications (Zhang et al., 2015; Vylomova et al., 2016) have already introduced the subword feature and achieved a remarkable result. Therefore, in our task, we also treat subword as an important feature.

Multitask Learning (Caruana, 1997; Zhang and Yang, 2017) is a method to train a learning model with different targets. When applying to multitask learning, the model could learn to extract features that are meaningful for both tasks and thus often lead to a more robust result. In this task, our goal is to identify the city of the given tweet. It is worth noticing that there does exist some relations between different cities. For example, two cities can locate within the same country, share the same time zone, or be closer than a specific distance. We believe using the hierarchical relation between cities could enable the model to learn extra information and thus improve the inference ability. Therefore, we introduce the joint training method into our model in order to take the relation between cities into consideration.

In the remainder of this paper, we will introduce related works in section 2. The problem definition and the detail of our proposed model will be described in sections 3, 4 respectively. In section 5, we will introduce the W-NUT 2016 Geo-tagging task and the in-depth analysis to illustrate the pros and cons of our proposed model.

## 2. Related Work

Location predicting has been studied for decades, but most of the work focuses on predicting a user's location. Recently, with the help of deep neural network, analyzing pure text is more feasible and thus researchers start trying to predict the location for a post, such as a tweet. In the following section, we will introduce the related location prediction tasks for user and post respectively.

For location inference of users, one well known approach is to infer the location from a graph structure (Backstrom et al., 2010; Davis et al., 2011; Li et al., 2012b,c; Jurgens, 2013; Rout et al., 2013; Compton et al., 2014; Kong et al., 2014; Jurgens et al., 2015). In these approaches, the main assumption is that friends will be very likely to live in the same location. Therefore, we could predict a user's location based on his relationship to other users. Among these works, Backstrom et al. (2010) is the first one noticing the interaction between geographical information and social relationship. They carefully examined the interaction and proposed a maximum likelihood approach to identify a user's location given the geographic information of the user's friends. Davis et al. (2011) built a following-follower network on Twitter and inferred a user's location based on a voting mechanism with three adjusting parameters. Li et al. (2012c) applied a Gaussian distribution to model a node's (friends or tweets) location as well as its influence scope. This network was then used to predict a user's location by maximizing the probability of building edges between the user and its friends or tweets. Li et al. (2012a) further extended the model to capture the property of a user having multiple related locations such as the home location as well as the college location. Their model is a revised version of Latent Dirichlet Allocation (LDA) model where the latent variables are locations. Rout et al. (2013) formulated the problem as a classification task and solved it by applying Support Vector Machine (SVM) with the features extracted from a Twitter's follower-based network. Jurgens (2013) extended label propagation method with the spatial property. As a semi-supervised learning method, spatial label propagation could iteratively inference all the user's location starting from only a few ground truth data. SPOT (Kong et al., 2014) took the social relation as a continuous feature instead of a binary feature (friends or not) by measuring the social closeness. The authors also introduced a confidence-based iteration method to overcome the data sparsity problem. Compton et al. (2014) formulated the social network geo-location inference task as a convex optimization problem and applied a total variation-based algorithm to solve it. These works rely on the information behind the social network and hence building a user relationship network is inevitable. This becomes a limitation if we want to work on data other than social media.

Another kind of method focuses on predicting using content and metadata provided by the user (Cheng et al., 2010; Eisenstein et al., 2010; Chandra et al., 2011; Roller et al., 2012; Mahmud et al., 2014). In Eisenstein et al. (2010)'s work, they presented a generative model to capture the relation between latent topics and geographical regions as they found that high-level topics such as “sport” and “entertainment” are rendered differently according to different location. Chandra et al. (2011) utilized only the content information but instead of using only a user's tweets, they augmented it with the replied tweets from other users by assuming that a reply tweet would have the same topic as the original tweet. A probability distribution model which could capture the relation between terms and locations was then applied to predict a user's location based on the corresponding augmented tweets set. Mahmud et al. (2014)'s work focused on building a hierarchical classification to integrate tweet contents, different categories of metadata, user's tweeting behaviors, and external location knowledge such as a geographic gazetteer dictionary. They also examined the impact of frequently traveling users and found that these users usually introduce noise into the model. This lead to a conclusion that eliminating frequently traveling users could improve the prediction accuracy. Roller et al. (2012) proposed an information retrieval method where the idea was to build a grid on the earth and then generate reference documents for each grid by selecting the location-related documents from training set. To overcome the problem of uniform grids, they constructed the grid using a k-d tree algorithm to dynamically adapt the grid size of the training data. Cheng et al. (2010)'s work focused on using purely content to predict the user's location with the assumption of location language difference. Although these approaches use mainly the content information, what they used is a bunch of posts provided by the user. As Cheng et al. (2010) and Chandra et al. (2011) revealed, given more posts, the accuracy would improve. This fact also suggests that predicting location for a single post is much difficult than for a user.

The tasks of predicting the location for a post were proposed much recently. After Han et al. (2016) built the dataset from Twitter and then proposed a shared task, researchers started digging into it. There are several approaches proposed in the shared task. Chi et al. (2016) applied a Naive Bayes classifier on many selected features, including location-indicative words, user meta data and so on. CSIRO (Jayasinghe et al., 2016) designed an ensemble method that incorporated heuristics, time zone text classifiers and an information retrieval approach. Miura et al. (2016) proposed a variant version of FastText Model which can take user's meta data into account. After the shared task, Huang and Carley (2017) designed a model with the help of the CNN layer. Lau et al. (2017), on the other hand, proposed the DeepGeo which utilized the character-level recurrent convolutional network to further capture the subword feature within the tweets. Most of these works tried to apply the deep learning framework to capture the language difference among the tweets. As we can see, with the help of deep learning framework, though we only have limited information in a single post, the result is still improving year by year.

## 3. Problem Definition

We use a bold capital letter to represent a matrix (e.g., **A**), a bold lowercase letter to represent a vector (e.g., **a**), and a normal lowercase letter to represent a scalar (e.g., a). Furthermore, a tweet which consists of *n* words is represented as **S** = {**w**_1_, **w**_2_, ··· , **w**_*n*_} where **S** is the tweet matrix and **w** is the word embedding. A tweet could also be represented as a sequence of *m* characters **C** = {**c**_1_, **c**_2_, ···**c**_*m*_} where **C** is the character matrix and **c** is the character embedding. Other general naming conventions are provided in Table 1.

**Table 1:** Notations and naming convention.

**Symbols**	**Definitions**
**w**	A word embedding $\in {\mathbb{R}}^{d_{w}}$.
**S** = {**w**_1_, **w**_2_, …, **w**_*n*_}	A text matrix consists of *n* word embeddings. The dimension is ${\mathbb{R}}^{n\times d_{w}}$.
**c**	A character embedding $\in {\mathbb{R}}^{d_{c}}$.
**C** = {**c**_1_, **c**_2_, …, **c**_*m*_}	A character matrix consists of *m* character embeddings. The dimension is ${\mathbb{R}}^{m\times d_{c}}$.
**E**	Embedding matrix.
**H**, **M**, **h**	hidden matrix and hidden vector.
**v**	Output vector.
**W**	Trainable weight matrix.
**b**	Trainable bias matrix.
*m*, *n*	Sequence length.
*d*	Dimension.
**y**_*city*_, **y**_*country*_	True city labels and true country labels.
${\text{y}}_{city}^{\prime }$, ${\text{y}}_{country}^{\prime }$	Predicted city labels and predicted country labels.

With the notation provided above, the problem definition could be described as:

**Table:** 

Problem Definition 1.	
**Given:**	A tweet and its corresponding representations, **S** and **C**.
**Predict:**	The label of the given tweet. This could be either *y*_*city*_ or *y*_*country*_.

## 4. Location Prediction for Tweets

In this chapter, we describe our model in several steps. We first introduce the high level architecture of our model. Then describe its separate modules, multi-head self-attention mechanism, subword features, and joint training method.

### 4.1. Model Overview

**Figure 1:**
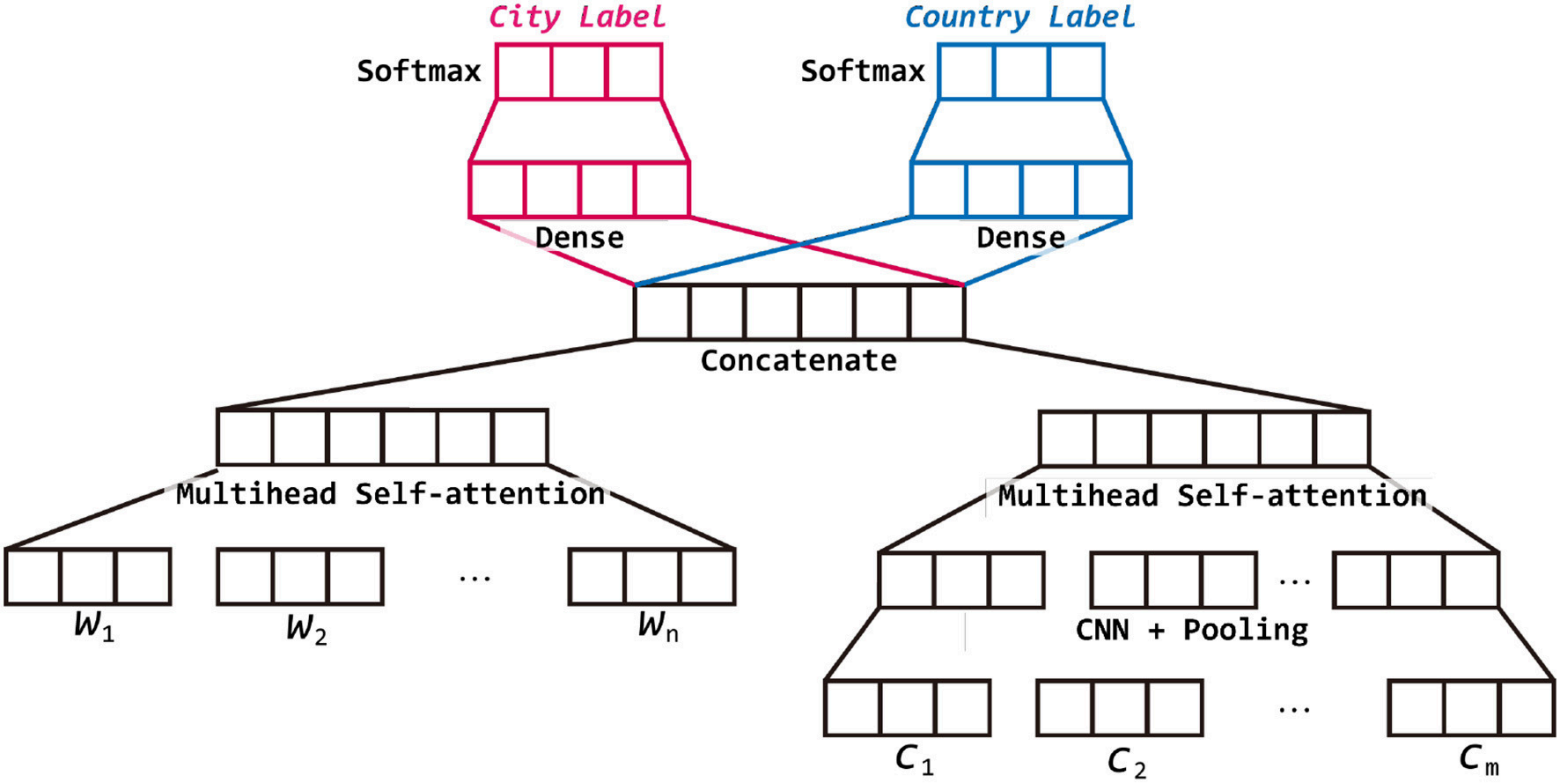
Overview of our proposed model.

As shown in Figure 1, the proposed model contains several small modules, but can be mainly separated into two parts including text representation and joint training. The text representation module consists of word representation and character representation. Both of the representation are encoded by multi-head self-attention layer but for character representation, we further use a CNN layer and pooling layer first to reduce the dimension and extract meaningful information. The word representation and character representation are then concatenated as a vector which represents the given tweet. In the second module, to utilize the relation between cities, we use the same concatenated vector but two different output layers to predict the country and city at the same time. However, the country classification is used only for training. In the testing phase, we use only the city part of the model for prediction.

### 4.2. Multi-Head Self-Attention Mechanism

The multi-head self-attention model is proposed by Vaswani et al. (2017) and is designed for language translation task. Here, we introduce the multi-head self-attention model as a module for text representation.

#### 4.2.1. Self-Attention

Let's start by defining the self-attention layer. In the normal attention mechanism, the model usually takes three inputs, a query **Q**, a key **K**, and a value **V**, where **Q** and **K** are used to compute the weights for **V**. The formal definition (Vaswani et al., 2017) is written as follows:

(1) $$\begin{array}{l} Attention\left(\text{Q},\text{K},\text{V}\right)=Softmax\left(\frac{\text{Q}{\text{K}}^{T}}{\sqrt{d}}\right)\text{V} \end{array}$$

where *d* is the word embedding dimension. However, in the self-attention layer, all the three inputs are the same matrix **S**, the text matrix {**w**_1_, **w**_2_, ··· , **w**_*n*_}, where **S** ∈ ℝ^*n*×*d*^, ${\text{w}}_{i}\in {\mathbb{R}}^{1\times d}$, *n* is the text length, and *d* is the word embedding dimension. By definition, the self-attention is:

(2) $$\begin{array}{l} \begin{array}{c} SelfAttn\left(\text{S}\right)=Attention\left(\text{S},\text{S},\text{S}\right)\\ \;=Softmax\left(\frac{\text{S}{\text{S}}^{T}}{\sqrt{d}}\right)\text{S} \end{array} \end{array}$$

However, we can make it clearer by defining it in word level. The **h**_*i*_ below is the transformation of **w**_*i*_ by weighted sum over the sentence.

(3) $$\begin{array}{l} \text{H}=SelfAttn\left(\text{S}\right)\\ \;=\left\{{\text{h}}_{1},{\text{h}}_{2},\cdot \cdot \cdot \;,{\text{h}}_{n}\right\} \end{array}$$

where **H** ∈ ℝ^*n*×*d*^ and ${\text{h}}_{i}\in {\mathbb{R}}^{1\times d}$. **h**_*i*_ is computed as follows:

(4) $$\begin{array}{l} {\text{h}}_{i}=\overset{n}{\underset{j=1}{\sum }}\alpha_{ij}\left({\text{w}}_{j}\right) \end{array}$$

α_*ij*_ is the weight for each **w**_*j*_ and is computed by the softmax function with the scaling term $\sqrt{d}$:

(5) $$\begin{array}{l} \alpha_{ij}=\frac{e^{{\text{w}}_{i}\cdot {\text{w}}_{j}^{T}/\sqrt{d}}}{\sum_{k=1}^{n}e^{{\text{w}}_{i}\cdot {\text{w}}_{k}^{T}/\sqrt{d}}} \end{array}$$

**Figure 2:**
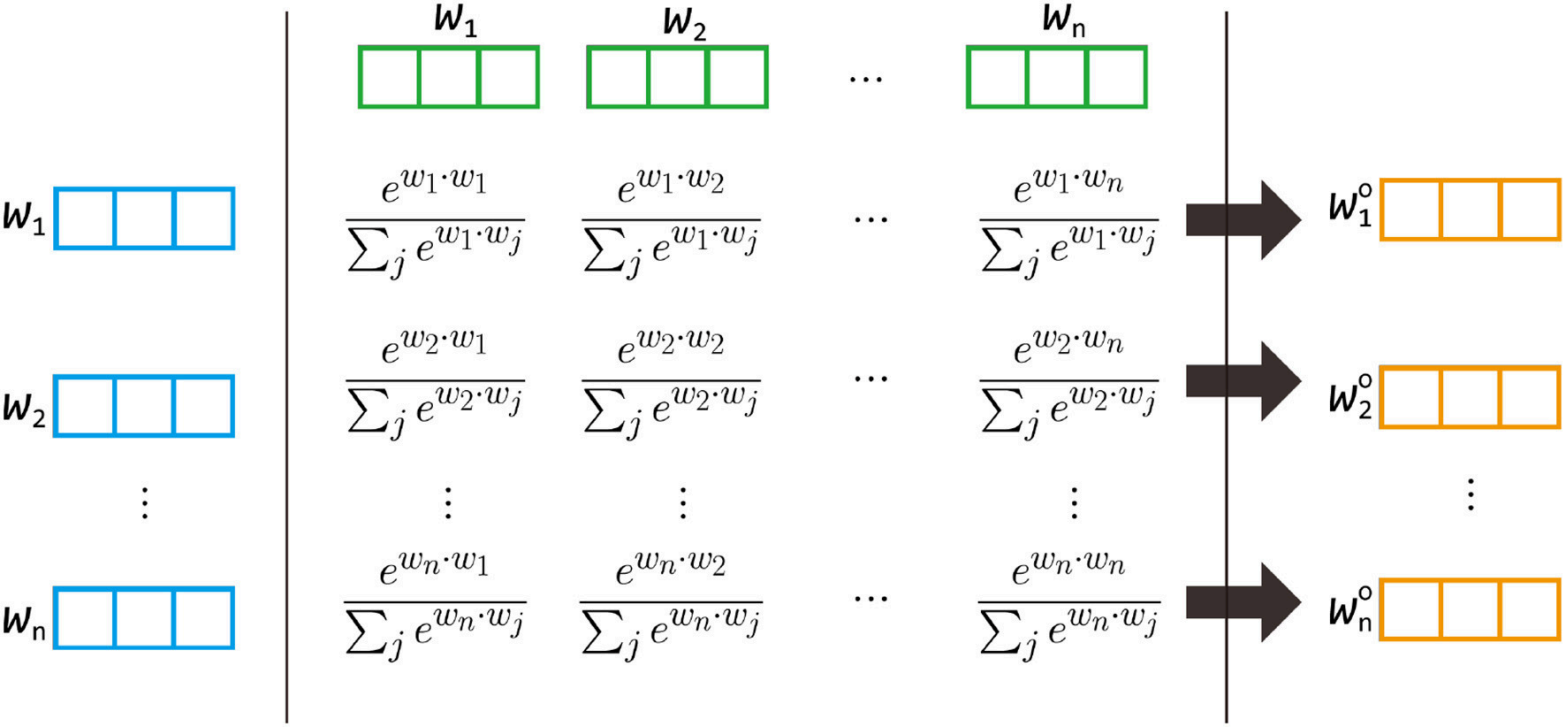
The idea of the multi-head self-attention model. ${\text{w}}_{i}^{o}$ will be reconstructed by {**w**_1_, *w*_2_…, **w**_*n*_} with the corresponding weights computed from **w**_*i*_ and {**w**_1_, **w**_2_…, **w**_*n*_}. Notice that the formula of the weight is actually $\frac{e^{{\text{w}}_{i}\cdot {\text{w}}_{j}/\sqrt{d}}}{\underset{j}{\sum }e^{{\text{w}}_{i}\cdot {\text{w}}_{j}/\sqrt{d}}}$. The figure is used to illustrate the idea of weights so $\sqrt{d}$ is not listed.

We also illustrate this idea in Figure 2. In this figure, we first use both the top part (green) and the left part (blue) to compute the corresponding weight for each cell. Equation (5) tells us that we need to normalize each row so the sum over each row is one. Then for each row, the new vector is constructed by summing up the vector in the top side (blue) multiplying by the corresponding weight.

#### 4.2.2. Multi-Head Self-Attention

In the above definition, the attention mechanism is performed only once, resulting only a single aspect vector. To equip the model with the power to learn multiple aspects information, a multi-head self-attention mechanism is proposed. In multi-head self-attention mechanism, we first apply a linear transformation **W** on **S**, producing **S**′ = **SW** where **S**′ ∈ ℝ^*n*×*d*′^, **S** ∈ ℝ^*n*×*d*^ and **W** ∈ ℝ^*d*×*d*′^. Notice that *d*′ < *d*, which means we are reducing the dimension. We then apply the self-attention model on **S**′:

(6) $$\begin{array}{l} {\text{H}}^{\prime }=SelfAttn\left({\text{S}}^{\prime }\right) \end{array}$$

The idea of multi-head self-attention mechanism is to perform the above work *h* times and then concatenate the resulting *h* vectors together. This gives the model the capability of learning *h* kinds of information. This functionality is defined as follows if *h* is set up as $d^{\prime }=\frac{d}{h}$:

(7) $$\begin{array}{l} \text{M}=MultiHead\left(\text{S}\right)\\ \;=Concat\left({\text{H}}_{1}^{\prime },{\text{H}}_{2}^{\prime },\cdot \cdot \cdot \;,{\text{H}}_{h}^{\prime }\right)\\ \;=Concat\left(SelfAttn\left({\text{S}}_{1}^{\prime }\right),SelfAttn\left({\text{S}}_{2}^{\prime }\right),\cdot \cdot \cdot \;,SelfAttn\left({\text{S}}_{h}^{\prime }\right)\right)\\ \;=Concat\left(SelfAttn\left(\text{S}{\text{W}}_{1}\right),SelfAttn\left(\text{S}{\text{W}}_{2}\right),\cdot \cdot \cdot \;,SelfAttn\left(\text{S}{\text{W}}_{h}\right)\right) \end{array}$$

As we can see, after performing the multi-head self-attention mechanism, the shape of the output matrix **M** is still ℝ^*n*×*d*^ which is the same as the input matrix **S**. Therefore, we could further apply the residual network (He et al., 2016) on the multi-head self-attention model. We revised Equation (7) as follow:

(8) $$\begin{array}{l} \text{M}=MultiHead\left(\text{S}\right)\\ \;=Concat\left({\text{H}}_{1}^{\prime },{\text{H}}_{2}^{\prime },\cdot \cdot \cdot \;,{\text{H}}_{h}^{\prime }\right)+\text{S} \end{array}$$

The idea of residual network is to add the input vector to the output vector. Since the original model is to learn a mapping function *F*(**x**), we change it to *F*′(**x**) = *F*(**x**) − **x**. The output **y** = *F*′(**x**) + **x** will still be the same but we could compute the gradient from the residual path and then reduce the gradient vanishing problem.

#### 4.2.3. Position-Wise Feed-Forward Network

The position-wise feed-forward network is introduced as the function of fully connected layer after the multi-head self-attention layer. The idea is to apply two linear transformations with a ReLU activation function on the input matrix. The mechanism could be described as follow:

(9) $$\begin{array}{l} {\text{M}}^{\prime }=FeedForward\left(\text{M}\right)\\ \;=\max\left(0,\;\text{M}\cdot {\text{W}}_{1}+{\text{b}}_{1}\right)\cdot {\text{W}}_{2}+{\text{b}}_{2} \end{array}$$

where ${\text{W}}_{1}\in {\mathbb{R}}^{d\times 4d}$, ${\text{W}}_{2}\in {\mathbb{R}}^{4d\times d}$ are transformation matrices and ${\text{b}}_{1}\in {\mathbb{R}}^{4d}$, ${\text{b}}_{1}\in {\mathbb{R}}^{d}$ are bias vectors. The transformation dimensions are suggested by Vaswani et al. (2017). We also apply the residual network here so Equation (9) will be revised as:

(10) $$\begin{array}{l} {\text{M}}^{\prime }=FeedForward\left(\text{M}\right)\\ \;=\left(\max\left(0,\;\text{M}\cdot {\text{W}}_{1}+{\text{b}}_{1}\right)\cdot {\text{W}}_{2}+{\text{b}}_{2}\right)+\text{M} \end{array}$$

where the last term **M** is identical to the input vector **M**.

#### 4.2.4. Position Encoding

One important thing that multi-head self-attention model can not model is the position relation between words. In RNN based model, the word order is well preserved since the output vector is calculated step by step. In the CNN based model, the word order is somehow preserved because it works on extracting information from the n-grams. However, the multi-head self-attention model utilizes weighted sum over the sequence of vectors where word order is totally ignored. Therefore, to fix this problem, Vaswani et al. (2017) introduced an idea of injecting the position information into the word vector. As a result, we build a position embedding matrix **E**_*position*_ (Gehring et al., 2017) and add the corresponding position vector to the word vector. **E**_*position*_ works as a word embedding layer which turns a position into a vector. This could be described as the following Equation:

(11) $$\begin{array}{l} {\text{w}}_{i}^{\prime }={\text{w}}_{i}+{\text{E}}_{position}\left[i,:\right] \end{array}$$

where **w**_*i*_ is the *i*-th word from **S** and *i* is the position. By introducing the position embedding, we expect that **E**_*position*_ can learn how to represent the position information. For example, one of the vector *e*_*position*1_ in **E**_*position*_ could learn the meaning of the first word. If we add *e*_*position*1_ to a word embedding, then the resulting vector should contain the information of the position (first word) and the meaning of the word. The position encoding is applied in the beginning of the whole model. As a result, after obtaining **w**′, we replace **S** by $\left\{{\text{w}}_{1}^{\prime },{\text{w}}_{2}^{\prime },\cdot \cdot \cdot \;,{\text{w}}_{n}^{\prime }\right\}$.

#### 4.2.5. Text Representation

In our model, we stack the multi-head self-attention layer and the position-wise feed-forward network twice. Notice that after passing through these layers, the output is still a matrix of ℝ^*n*×*d*^. In order to get the text representation, we need to reduce the output matrix into a one dimensional vector. As a result, we simply sum up alone the sequence dimension *n*, producing the text representation as follow:

(12) $$\begin{array}{l} {\text{v}}^{text}=\overset{n}{\underset{i=1}{\sum }}{\text{M}}^{\prime }\left(i,\;:\right) \end{array}$$

the resulting **v**^*text*^ ∈ ℝ^*d*^.

### 4.3. Subword Feature

The idea of adding a subword feature is to infer the meaning of the low frequency word. Though the most simple way here is to apply the above model directly to the character level sequence, it will be infeasible since the complexity of multi-head self-attention model is *O*(*n*^2^ · *d*), where *n* is the sequence length. Although the maximum number of characters allowed in Twitter is only 140, it will still cause a huge computation bottleneck in our model. Therefore, we first apply one-dimensional convolutional neural network to extract the n-gram information, and then use maximum pooling over a small window to extract meaningful information and at the same time reduce the sequence length. The detailed procedure is described in the following paragraph.

Given the character matrix **C**, we first apply a convolutional neural network layer to it. Each element of the resulting matrix **H**^*conv*^ could be described as:

(13) $$\begin{array}{l} h_{ij}^{conv}=\text{C}\left(i-k:i+k,\;:\right)*{\text{W}}_{j}^{c}+{\text{b}}_{j}^{c} \end{array}$$

where, * is the convolution operator, *k* is half of the kernel size, *i* is the index of the character sequence ranging from 1 to the character length *m*, and *j* is the index of the filter ranging from 1 to the number of filters *f*.

After this, we apply the maximum pooling with a window size equal to the kernel size 2*k* and then slide 2*k* − 1 to next step. Therefore, the element of the resulting matrix **H**^*pool*^ is given by:

(14) $$\begin{array}{l} \begin{array}{c} h_{ij}^{pool}=\max\left({\text{H}}^{conv}\left(l-k:l+k,\;j\right)\right),\\ l=i\cdot \left(2k-1\right),\;i=1,\cdot \cdot \cdot \;,\frac{m}{2k-1} \end{array} \end{array}$$

As we can see, the first dimension reduces from *m* to $\frac{m}{2k-1}$ and thus **H**^*pool*^ is ${\mathbb{R}}^{\frac{m}{2k-1}\times f}$. We then apply the multi-head self-attention model on **H**^*pool*^ and get **v**^*char*^ ∈ ℝ^*f*^ as:

(15) $$\begin{array}{ll} {\text{M}}^{char} & =MultiHead\left({\text{H}}^{pool}\right) \end{array}$$

(16) $$\begin{array}{ll} {\text{M}{\;}^{\prime }}^{char} & =FeedForward\left({\text{M}}^{char}\right) \end{array}$$

(17) $$\begin{array}{ll} {\text{v}}^{char} & =\overset{\frac{m}{2k-1}}{\underset{i=1}{\sum }}{\text{M}{\;}^{\prime }}^{char}\left(i,\;:\right) \end{array}$$

### 4.4. Joint Training

The **v**^*text*^ and **v**^*char*^ are then concatenated as the tweet representation vector.

(18) $$\begin{array}{l} {\text{v}}^{tweet}=Concat\left({\text{v}}^{text},{\text{v}}^{char}\right) \end{array}$$

where **v**^*tweet*^ ∈ ℝ^*d*+*f*^.

By applying different transformation **W**_*city*_ and **W**_*country*_, we could get two different vector **v**_*city*_ and **v**_*country*_ for prediction.

(19) $$\begin{array}{ll} {\text{v}}_{city} & ={\text{v}}^{tweet}{\text{W}}_{city} \end{array}$$

(20) $$\begin{array}{ll} {\text{v}}_{country} & ={\text{v}}^{tweet}{\text{W}}_{country} \end{array}$$

where $W_{city}\in {\mathbb{R}}^{\left(d+f\right)\times m_{city}}$, $W_{country}\in {\mathbb{R}}^{\left(d+f\right)\times m_{country}}$, ${\text{v}}_{city}\in {\mathbb{R}}^{m_{city}}$, ${\text{v}}_{country}\in {\mathbb{R}}^{m_{country}}$, and *m*_*city*_, *m*_*country*_ are city size and country size respectively.

We then apply the softmax function to get the probability for each city $p_{city}^{l}$ and each country $p_{country}^{l}$.

(21) $$\begin{array}{ll} p_{city}^{l} & =\frac{e^{v_{city}^{l}}}{\sum_{k=1}^{m_{city}}e^{v_{city}^{k}}} \end{array}$$

(22) $$\begin{array}{ll} p_{country}^{l} & =\frac{e^{v_{country}^{l}}}{\sum_{k=1}^{m_{country}}e^{v_{country}^{k}}} \end{array}$$

where $v_{city}^{l}$ is the *l*-th element of **v**_*city*_ and $v_{country}^{l}$ is the *l*-th element of **v**_*country*_.

The prediction of city $y_{city}^{\prime }$ and country $y_{country}^{\prime }$ is the label with the highest probability.

(23) $$\begin{array}{ll} y_{city}^{\prime } & =\underset{l}{\text{argmax}}\left(p_{city}^{1},p_{city}^{2},\cdot \cdot \cdot \;,p_{city}^{m_{city}}\right) \end{array}$$

(24) $$\begin{array}{ll} y_{country}^{\prime } & =\underset{l}{\text{argmax}}\left(p_{country}^{1},p_{country}^{2},\cdot \cdot \cdot \;,p_{country}^{m_{country}}\right) \end{array}$$

In our task, we have two kinds of labels; city and country. As we could see here, some cities are actually in the same country and thus shared some common information. Therefore, we proposed the joint training framework for modeling city and country at the same time. Since we use cross-entropy as our loss function, the joint learning loss function is as follows:

(25) $$\begin{array}{l} Loss=-\overset{m_{city}}{\underset{l=1}{\sum }}y_{city}^{l}\log p_{city}^{l}-\overset{m_{country}}{\underset{l=1}{\sum }}y_{country}^{l}\log p_{country}^{l} \end{array}$$

where $y_{city}^{l}$ and $y_{country}^{l}$ are binary indicators (0, 1) which will give 1 only if the label is the correct one.

## 5. Experiments and Results

In this section, we describe the experiment on the W-NUT 2016 Geo-tagging task^1^ and some benchmark approaches for comparison. Different metrics are utilized in this experiment to provide insights from different aspects.

### 5.1. Data

We directly use the geolocation prediction shared task dataset (Han et al., 2016) in our experiment. Though they provide two tasks, predicting locations for tweets and users, we only focus on the tweet prediction part. The dataset is collected from 2013 to 2016 by Twitter Streaming API. Besides, only tweets whose language are identified by Twitter as English are retained. Due to the limitation of Twitter policy, the dataset provides only the ID of the collected tweets instead of the original tweets and the corresponding information. As a result, although the dataset provides 12M and 10k tweets for training and developing, we could only collect about 8M and 8k tweets respectively since users could delete the tweets they posted and thus some tweets are no longer available. However, the testing data containing 10k tweets is shared comprehensively so comparing with the previous benchmark is possible. The detail statistic information is provided in Table 2.

**Table 2:** Statistic of the W-NUT geo-tagging task dataset.

**Data**	**Amount**
Training Set	8,492,598
Validation Set	7,214
Testing Set	10,000
City Label	3,362
Country Label	175

## 6. Evaluation Metrics

There are three metrics adopted in the W-NUT 2016 Geo-tagging task, including one hard metric and two soft metrics. The first way is the classification **accuracy** over the city prediction. This is regarded as a hard metric because there is no tolerance for a wrong prediction. The distance-based metric, on the other hand, is regarded as a soft metric as it measures the distance between the true value and the predicted value. Therefore, only a wrong prediction with large error will be penalized a lot. Two distance-based metric is utilized, **median error distance** and **mean error distance**. Given the evaluation result *R* = *d*_1_, *d*_2_, ··· , *d*_*n*_, where *d*_*n*_ is the error distance (kilometers) between the predicted and the standard geographic coordinate, median error distance and mean error distance are computed as follows:

(26) $$\begin{array}{l} MedianErrorDistance=Median\left(R\right) \end{array}$$

(27) $$\begin{array}{l} MeanErrorDistance=Mean\left(R\right) \end{array}$$

### 6.1. Benchmark Model

Several benchmarks are selected for comparison. Since the original dataset provides metadata like user specified location description, timezone, self-introduction, and so on, most of the work utilizes all of these information in their model. However, our proposed model focuses on modeling the information behind the content itself. As a result, we implemented the text-content-only version for these models by removing the modules or layers that deal with the metadata. In the following section, we describe several benchmarks and how we remove the metadata-related modules.

#### 6.1.1. DeepGeo

DeepGeo (Lau et al., 2017) utilized the character-level recurrent convolutional network (Lai et al., 2015) for text modeling. In the recurrent convolutional network, the character matrix is passed through a bi-directional LSTM layer, producing a hidden state matrix. The hidden state matrix is then passed through a CNN layer followed by a max-over-time pooling layer to generate the subword features. After acquiring the subword features, a attention network is applied to merge the subword feature matrices into a single vector. In addition to the text representation module, deepgeo introduce a RBF network for modeling the time-related features, such as Tweet creation time and account creation time. All of these vectors including the text representation and meta feature are then concatenated and go through two dense layers for classification. To understand the model's ability of handling the text information, we remove the layers other than the character-level RCNN.

#### 6.1.2. FUJIXEROX

This approach is proposed by FUJIXEROX (Miura et al., 2016), one of the participated team in W-NUT 2016 Geo-tagging task. This model is a variant version of the original FastText Model (Bojanowski et al., 2016). The idea is to represent a word by the sum of its n-gram embeddings. Therefore, for a out-of-vocabulary word, the model could still inference its word vector according to the subword features (n-gram). FUJIXEROX applied FastText model on not only the tweet text but also the user specified location and the user profile description. The three feature vectors and the time zone embedding vector are then concatenated then passed into a dense layer for prediction. To have it use only the text information from tweets, the metadata features are removed and the resulting model is a supervised FastText model.

#### 6.1.3. CNN Model

A CNN-based model is provided by Huang and Carley (2017). Their approach is to use a CNN layer (Kim, 2014) for modeling the tweet content, the user profile description, the user specified location, and the user name. Then, these four vectors are concatenated with four one-hot vectors, tweet language, user language, time zone and the tweet creation time. The concatenated vector is then passed through a dense layer and form a classifier. Unlike the previous two approaches, this task is performed on a self-built dataset. Therefore, we implemented this approach for comparison. The model after removing the metadata feature is actually a CNN model.

#### 6.1.4. CSIRO

Jayasinghe et al. (2016) utilize ensemble approaches to overcome the weakness of each component. They also handle many kinds of metadata and integrate them with external information like gazetteer, IP-Lookup table, and so on. They then apply these features to label propagation approach, information retrieval approach, and text classification approach. By examining different ensemble strategies, they found that the full cascade one outperforms the other strategies. As their approach heavily relies on the metadata, we only list it as a reference.

#### 6.1.5. Naive Bayes Methods

This method is proposed by Chi et al. (2016) with the use of naive Bayes methods on many selected features. However, only features extracted from text data are considered such as location-indicative words, hashtags and so on.

### 6.2. Experiment Setting

The parameters used for our model are listed in the Table 3. If we combine a multi-head self-attention layer and a feed-forward layer as an attention layer, then the stack number 2 means we stack two attention layers and produce a series of layers as multi-head self-attention, feed-forward, multi-head self-attention, feed-forward. We use Adam (Kingma and Ba, 2014) for optimization. The model is trained for 10 epochs and then the one with the best validation result is kept for testing. To compute the distance error, we didn't use the model to predict latitude and longitude. However, we map the predicted city into the corresponding latitude and longitude and then take it as our prediction for the geographic coordinate. For example, if the predicted city label is “los angeles-ca037-us,” we search on GeoNames^2^ by the query “Los Angeles US” (city name and country name). The geographic coordinate (N 34°3′ 8′′, W 118°14′ 37′′) is used as the predicted result.

**Table 3:** Detail of the Parameter Setting. Setting_2_ is to use only single-head self-attention model. Setting_3_ is trained without country label.

**Network**	**Parameter**	**Size_**1**_**	**Size_**2**_**	**Size_**3**_**
Overall	Batch Size	512	512	512
	Epochs	10	10	10
	Dropout	0.3	0.3	0.3
	Learning Rate	0.0005	0.001	0.001
	Min Word Frequency	10	10	10
Text	Max Length	30	30	30
	Heads	10	1	2
	Stack Number	2	2	2
	$d^{E_{word}}$	200	200	200
	*d*^*h*^	200	200	200
Character	Max Length	140	140	140
	Heads	8	1	2
	Stack Number	2	2	2
	$d^{E_{char}}$	100	100	100
	*d*^*h*^	100	100	100
	CNN filter size	3, 4, 5, 6, 7	3, 4, 5	3, 4, 5
	filter number	64	64	64

*In the pre-processing step, words that appear less then the min word frequency times will be turned into <UNK> token. Max length means the maximum sequence length so exceeding words will be removed. If the text sequence doesn't meet the max length, we will pad <nan> in front of the text*.

### 6.3. Result

The results are listed in Table 4. We separate the result into two sections because we mainly focus on the setting without metadata. Within this setting, our model outperforms all the other models according to Acc_1_, the accuracy of the city. FUJIXEROX's fastText model performs relatively well in both of the distance measurements but our proposed approach is competitive. For the rest of the methods, Proposed Method_1_ outperforms DeepGeo 8.98% and 22.07%, CNN 14.35% and 27.49%, Naive Bayes 18.08% and 43.09% in mean error distance and median error distance respectively. This phenomenon suggests that our proposed method could better capture the location relation. To better understand the behavior of the model, we try to examine the country-wise prediction. Here, we turn a city label into a country label by extracting the country from the city label. For example, the country label for “los angeles-ca037-**us**” is “us.” We then compute the accuracy and report it also in Table 4 as **Acc**_2_. As we can see, there is no huge difference between Acc_2_ which suggests that our proposed method gives a closer city prediction within the same country. Let's move to the setting with metadata. The results are reported by their papers so part of the table is missing. It is, however, easy to see that the results of using metadata improve a lot. This is foreseeable since some of the metadata provide very strong information. For instance, the timezone feature basically acts as a geographic constraint. Not to mention that some users explicitly reveal their home location in the profile location description which becomes another metadata. As we state before, we focus on extracting the information from pure text content, so it is reasonable for us to ignore the metadata.

**Table 4:** Result of model using only text information. Acc_1_ means the accuracy of the city and Acc_2_ represents the accuracy of the country.

**Model**	**Acc_**1**_**	**Acc_**2**_**	**Mean**	**Median**
**Without Metadata**				
Naive Bayes^*^	0.146	–	5338.9	3424.6
FUJIXEROX	0.168	0.566	4441.5	**1900.5**
CNN Model	0.207	0.581	5106.8	2687.6
DeepGeo^+^	0.202	**0.597**	4805.5	2500.9
Proposed Method_1_	**0.218**	0.590	**4373.7**	1948.9
Proposed Method_2_	0.215	**0.597**	4449.2	1970.6
Proposed Method_3_	0.216	0.581	4697.2	2088.4
**With Metadata**				
FUJIXEROX	0.409	–	1792.5	69.5
DeepGeo	0.428	–	–	–
CSIRO	0.436	–	2538.2	74.7

* *Notice that the result of Navie Bayes is reported in Chi et al. (2016) where Acc_2_ is not available. ^+^Though the reported accuracy of Acc_1_ in Lau et al. (2017) is 0.217, our experiment gives only 0.202. The results with metadata are provided in the original paper and therefore some numbers are missing. The bold values mean the best performances for each column in the “without metadata” setting*.

When comparing different settings of our proposed method, we can see that Proposed Method_1_ performs the best. In Proposed Method_2_, where we set the head number to 1 and get the single-head self-attention model, the performance generally decrease in both Acc_1_ and the distance measurements. However, when reducing the head number to one, the training time also reduces a lot. In Proposed Method_3_, we tried to train the model without the country label. As we could see, the distance measurements increase, especially the mean error distance. This means that using country label could really help the model learn the geographic relation between cities.

### 6.4. Analysis

In this section, we analyze some cases by printing the attention weight matrix. We only focus on the word representation module of the Proposed Method_2_ since the multi-head attention model have several attention weight matrices and thus is hard to illustrate. Also, as the character representation module contains the CNN layer and pooling layer, it is hard to understand which subword feature is kept in the attention layer. Therefore, we focus on analyzing the word representation module to see what the Proposed Method_2_ learned.

**Figure 3:**
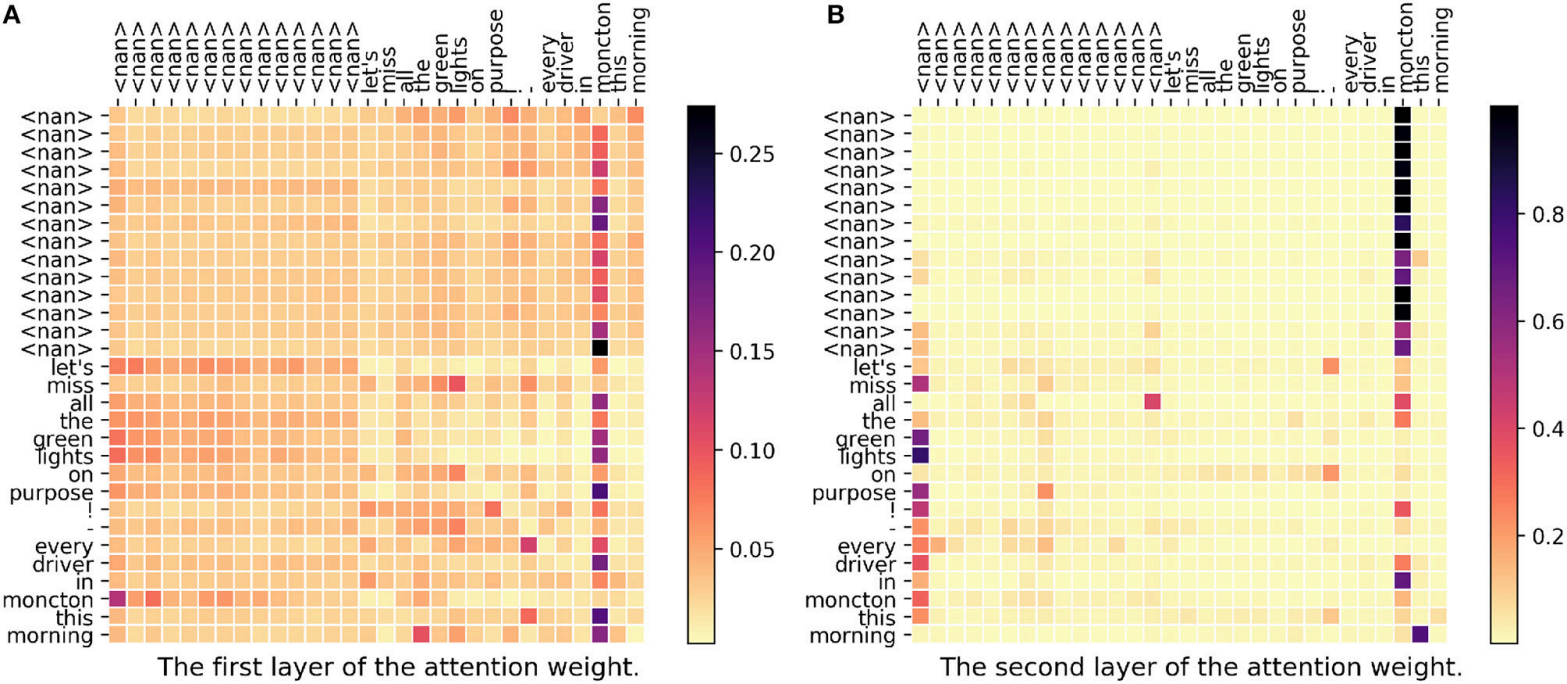
**(A,B)** The attention weight matrix of our model. <nan> is the padding term since the word length is <30. In this case, DeepGeo, CNN and the proposed method correctly predict **moncton-04-ca** but FUJIXEROX predicts **los angeles-ca037-us**.

In Figure 3, we can see there are two attention weight matrices because we have two stacked self-attention layers in our model. In the figure, each row represents a set of weights to construct the new vector. For example, in the last row where the word is “morning,” only “all” as well as “moncton” have higher weights. As we can see in both first layer and second layer, “moncton” get a very high weight in most of the words. Since this word reveals the location directly, DeepGeo, CNN and our proposed model give the correct prediction. FUJIXEROX, on the other hand, predicts **los angeles-ca037-us** and fails to give the correct label. Notice that in the first layer (Figure 3A), the weights of the first <nan> distribute evenly over the words. As a result, after the first layer, the vector of <nan> could be seen as a sentence representation. This is why in the second layer (Figure 3B), lots of the words also highly attend on the first column (<nan>).

**Figure 4:**
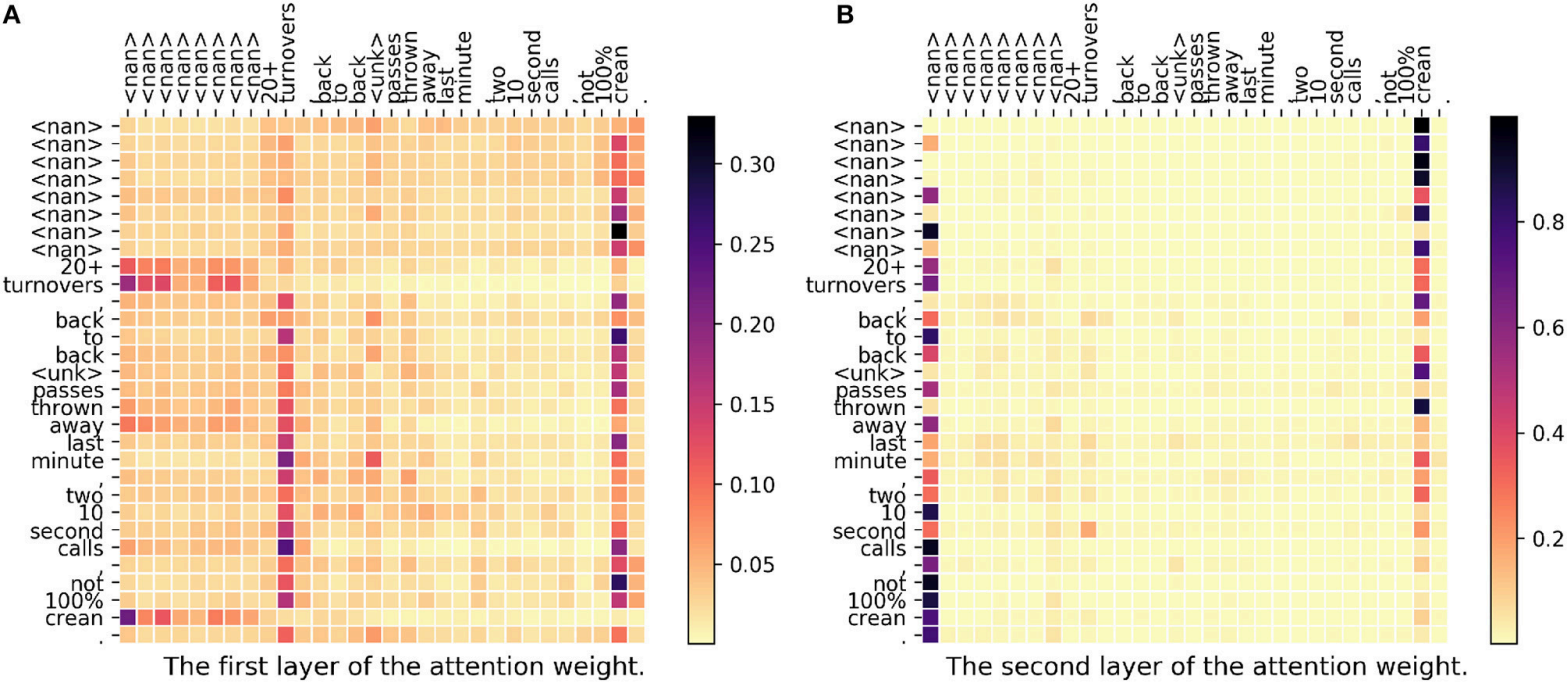
**(A,B)** The attention weight matrix of our model. <nan> is the padding term since the word length is <30. In this case, only our proposed method correctly predict **indianapolis-in097-us**. DeepGeo, CNN, and FUJIXEROX predict **city of london-enggla-gb**, **chicago-il031-us**, and **toronto-08-ca** respectively.

In Figure 4, since the topic is about basketball, “crean” then becomes a very important word. Actually, “crean” stands for a basketball coach, Tom Crean, in Indiana University. In the first layer, we could see that both “turnovers” and “crean” get a high weight meaning that the model successfully capture the relation between a basketball term “turnovers” and a person name “crean.” In the second layer, <nan> and “crean” get high weights. Notice that the vector of <nan> could also be seen as a sentence representation. As a conclusion for this case, our proposed model successfully captures the hidden relations and gives a correct prediction but DeepGeo, CNN, and FUJIXEROX all fail in this case.

**Figure 5:**
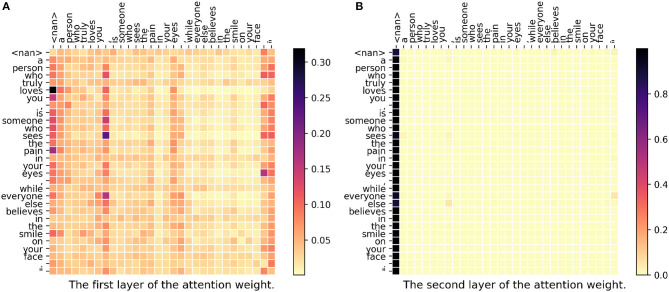
**(A,B)** The attention weight matrix of our model. <nan> is the padding term since the word length is <30. In this case, the correct city is **kisumu-07-ke** but DeepGeo, CNN, FUJIXEROX, and the proposed method predict **lagos-05-ng**, **lagos-05-ng**, **quezon city-ncrf2-ph**, and **kano-29-ng** respectively.

In Figure 5, all the four models fail to recognize the location correctly, since this post does not give any useful information. We could find that in the first layer, all the weights are similar. Also, in the second layer, all of the word attends on the first column (<nan>) where the vector does not contain any useful information. This means that our model could not find any meaningful and helpful information for prediction.

**Figure 6:**
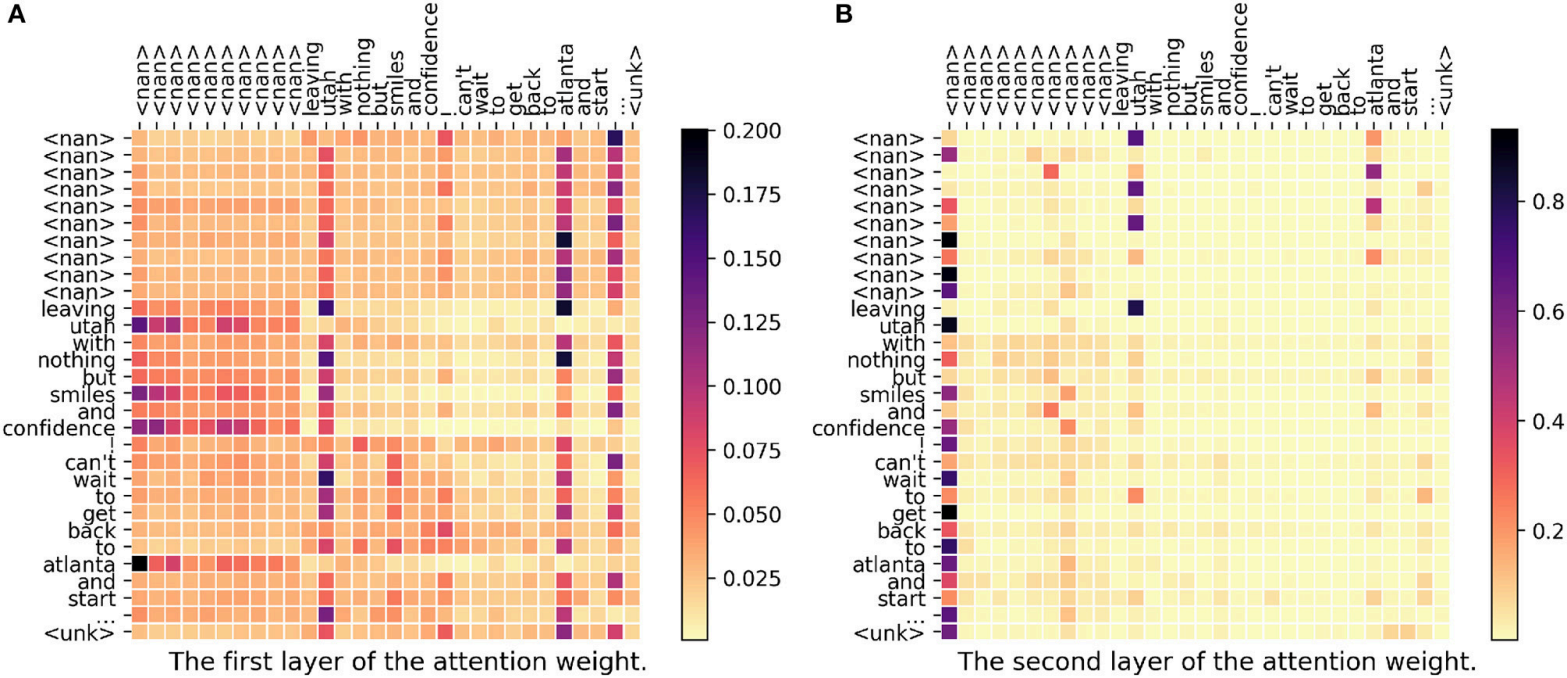
**(A,B)** The attention weight matrix of our model. <nan> is the padding term since the word length is <30. In this case, DeepGeo, CNN, and FUJIXEROX correctly predict **salt lake city-ut035-us** but our proposed method predicts **atlanta-ga121-us**.

In Figure 6, both DeepGeo, CNN, and FUJIXEROX predict the correct city, **salt lake city-ut035-us**, but our model predicts **atlanta-ga121-us**. When investigating the weight matrix, we can find that both “utah” and “atlanta” get high attention which somehow represents the two label **salt lake city-ut035-us** and **atlanta-ga121-us** respectively. This gives a controversial information to our model and thus in the end our model predicts the wrong label. In conclusion, our proposed model fails to capture the semantic meaning of “leaving.”

The above four cases give us a brief understanding of the behavior of our proposed model. Our model could capture the hidden relations between different terms. However, it is still suffering from understanding the semantic meaning of words so it gives wrong predictions sometimes. Generally, the information captured by the model is easy to understand and quite meaningful.

## 7. Conclusions

In this paper, we have proposed a new deep learning model to predict location for tweets. Our model integrates three key concepts, including multi-head self-attention mechanism, subword feature, and joint training technique with country label. The experiment on W-NUT geo-tagging task shows our model is competitive or better than the state-of-the-art methods w.r.t. different measurements. The analysis on attention weight matrix also illustrates that our model can capture the hidden relations between different words. In the future, we will further consider the semantic information of the sentences to better capture the meaning of the tweet.

## Author Contributions

C-YH designed the model, performed the experiments, analyzed the data, and wrote the paper. HT gave suggestions for the model as well as the experiments and wrote the paper. JH and RM contributed in the early discussion and the problem identification.

### Conflict of Interest Statement

The authors declare that the research was conducted in the absence of any commercial or financial relationships that could be construed as a potential conflict of interest.

## Footnotes

^1^https://noisy-text.github.io/2016/geo-shared-task.html

^2^https://www.geonames.org/
